# Children’s Non-symbolic and Symbolic Numerical Representations and Their Associations With Mathematical Ability

**DOI:** 10.3389/fpsyg.2018.01035

**Published:** 2018-06-25

**Authors:** Yanjun Li, Meng Zhang, Yinghe Chen, Zhijun Deng, Xiaoshuang Zhu, Shijia Yan

**Affiliations:** ^1^School of Developmental Psychology, Faculty of Psychology, Beijing Normal University, Beijing, China; ^2^National Innovation Center for Assessment of Basic Education Quality, Beijing Normal University, Beijing, China; ^3^Department of Psychology, Rutgers, The State University of New Jersey, New Brunswick, NJ, United States; ^4^China Aerospace Academy of Systems Science and Engineering, Institute of Information Control, China Aerospace Science and Technology Corporation, Beijing, China

**Keywords:** non-symbolic numerical representation, symbolic numerical representation, mapping, mathematical ability, mathematical development

## Abstract

Most empirical evidence supports the view that non-symbolic and symbolic representations are foundations for advanced mathematical ability. However, the detailed development trajectories of these two types of representations in childhood are not very clear, nor are the different effects of non-symbolic and symbolic representations on the development of mathematical ability. We assessed 253 4- to 8-year-old children’s non-symbolic and symbolic numerical representations, mapping skills, and mathematical ability, aiming to investigate the developmental trajectories and associations between these skills. Our results showed non-symbolic numerical representation emerged earlier than the symbolic one. Four-year-olds were capable of non-symbolic comparisons but not symbolic comparisons; five-year-olds performed better at non-symbolic comparisons than symbolic comparisons. This performance difference disappeared at age 6. Children at age 6 or older were able to map between symbolic and non-symbolic quantities. However, as children learn more about the symbolic representation system, their advantage in non-symbolic representation disappeared. Path analyses revealed that a direct effect of children’s symbolic numerical skills on their math performance, and an indirect effect of non-symbolic numerical skills on math performance via symbolic skills. These results suggest that symbolic numerical skills are a predominant factor affecting math performance in early childhood. However, the influences of symbolic and non-symbolic numerical skills on mathematical performance both declines with age.

## Introduction

### The Developmental Trajectories of Non-symbolic and Symbolic Representation Abilities

A variety of studies have suggested that animals and humans shared the capacity of non-symbolic representation (Wynn, 1992; Pica et al., 2004; Flombaum et al., 2005), which has been attributed to the so-called approximate number system (ANS) (Feigenson et al., 2004; Barth et al., 2005, 2006, 2008; Dehaene, 2011). The ANS system has three features. First, it is inherent and universal (Wynn, 1992; Pica et al., 2004; Flombaum et al., 2005); animals and humans share the system. Second, it represents quantities in an approximate way (Feigenson et al., 2004). Third, the precision of ANS system increases with age (Halberda et al., 2008). Correspondingly, there are three different characteristics for symbolic number representation system. First, it is an acquired system, it is affected by the language faculties (Pica et al., 2004; Xenidou-Dervou et al., 2015). Second, it represents quantities precisely (Izard and Dehaene, 2008; Mussolin et al., 2014). Third, with age, the system can manipulate increasingly larger range with higher accuracy (Halberda et al., 2008; Praet and Desoete, 2014).

Children’s non-symbolic skills emerge early and develop continuously over time (Barth et al., 2005, 2006, 2008; Halberda et al., 2008). Libertus et al. (2011) assessed non-symbolic skills with numbers range 4 to 15. They found that 4-year-olds were able to complete their non-symbolic comparison task. Toll et al. (2015) tested non-symbolic skills with a larger range of 1–100 and found the similar results in 4-year-olds. Wagner and Johnson (2011) assessed non-symbolic skills with numbers range 1–50. They found 3-year-olds performed above chance level in non-symbolic comparison task with numerosities 1–4. Many studies (Barth et al., 2005; Sasanguie et al., 2013; Hyde et al., 2014; Vanbinst et al., 2015) examined the non-symbolic comparison ability in 5-year-olds and older children. They found the skill kept developing during childhood, even till adulthood. Barth et al. (2005) found that adults were significantly more accurate than 5-year-old children in the non-symbolic comparison task.

Research showed symbolic skills emerged at 5 years old, before the start of formal schooling (Kolkman et al., 2013). Children were able to do symbolic representation task at age 5 (Gilmore et al., 2007). What makes them capable of symbolic numerical representations before formally learning numerical symbols? Some researchers (Gilmore et al., 2007) argued that children might pass the task with the help of their ANS. It is plausible that they converted symbolic Arabic numbers to non-symbolic numerosities. In other word, they had the mapping ability, which enabled the process of transforming non-symbolic representation and symbolic representation information into one another. Other researchers argued that informal mathematical activities help improve children’s symbolic skills (Skwarchuk et al., 2014; Berkowitz et al., 2015). Although 4- or 5-year-old children have not obtained mathematical education from school, they may have already been exposed to many informal mathematic activities, such as playing number board game, reading stories involved quantities, and so on. With so many possible exposures to mathematical knowledge, this study tries to explore whether children as young as 4 years old are able to represent and compare symbolic Arabic numbers.

The relationship between symbolic skills and non-symbolic skills has been discussed a lot in this field. Some researchers claim that non-symbolic and symbolic skills are separable (They adopted non-symbolic comparison and symbolic comparison tasks which are similar to tasks in our current study) (Kolkman et al., 2013). They rely on two distinct systems and do not share the same underlying ability (Xenidou-Dervou et al., 2015). Other researchers believed that both non-symbolic and symbolic comparison abilities, to some extent, relied on the ANS system (Chen and Li, 2014; van Marle et al., 2014). Furthermore, the majority of previous studies focused on the correlation between non-symbolic and symbolic representation skills (Castronovo and Goebel, 2012; Gobel et al., 2014). Most researchers believe there is a positive correlation between non-symbolic and symbolic skills (Kolkman et al., 2013; van Marle et al., 2014; Toll et al., 2015). Other researchers (Fazio et al., 2014) found no correlation between these two types of skills. The available evidence is not congruent, both distinctions and connections between symbolic and non-symbolic comparison abilities were reported. The development trajectories of these two are not very clear. Some tasks used by previous researchers were too difficult to detect children’s emerging numerical skills. For example, Xenidou-Dervou et al. (2015) assessed 5- and 6-year-olds’ non-symbolic and symbolic abilities by using approximate addition tasks, which were harder than comparison. In their task, children had to add the two quantities first and then to compare the numerosities. That is to say, their task also required children’s arithmetic ability at the same time. The present study used comparison tasks to test both symbolic and non-symbolic abilities. We aim to provide more comprehensive developmental trajectories of non-symbolic and symbolic capacities in preschoolers and young primary students.

### The Associations Between Numerical Representation Skills and Mathematical Ability

The association between non-symbolic representation and mathematical ability is not clear. Many studies showed positive correlations between non-symbolic representation skills and mathematical ability in children and adults (DeWind and Brannon, 2012; Libertus et al., 2012; Bonny and Lourenco, 2013). Libertus et al. (2012) assessed 3- to 5-year-olds’ non-symbolic comparison precision and mathematical ability. They found there was a significant positive correlation between the precision of non-symbolic task and mathematical achievement. Halberda et al. (2008) found similar results in older children. Furthermore, longitudinal data showed that non-symbolic skills in early childhood significantly predicted later mathematical abilities (Halberda et al., 2008; Mazzocco et al., 2011; Libertus et al., 2013). However, other researchers did not find positive correlations between non-symbolic representation skills and mathematical ability in children (Holloway and Ansari, 2009; Sasanguie et al., 2013) and adults (Inglis et al., 2011; Price et al., 2012). It appears that not all researchers consider that non-symbolic representation ability and mathematical ability are related. Therefore, the issue, whether the ability of non-symbolic representation play an important role in the development of mathematical ability or not, needs further explorations.

Researchers have reached a consensus about the relationship between symbolic skills and mathematical ability. That is, symbolic skills have a significant impact on mathematical ability. Bugden and Ansari (2011) found a significant positive correlation between symbolic comparison skills and mathematical ability in 1st and 2nd grade children from primary school. Toll et al. (2015) investigated children’s non-symbolic and symbolic comparison skills; they found that symbolic comparison skills were the most important predictor for mathematical ability. Similar results were also found from a longitudinal study (Kolkman et al., 2013).

Most empirical evidence supports the view that non-symbolic and symbolic comparison skills are foundations for advanced mathematical ability (Libertus et al., 2011; Castronovo and Goebel, 2012). van Marle et al. (2014) assessed non-symbolic, symbolic skills, and mathematics ability of 4-year-olds. They found the relation between non-symbolic skills and mathematics ability was completely mediated by children’s performance on the symbolic comparison task. Similar results were also found in 6-year-olds (Gobel et al., 2014). However, Fazio et al. (2014) assessed children of 10 years old, and they found symbolic and non-symbolic skills related to mathematics ability uniquely. Up to now, it still seems unclear how non-symbolic, symbolic comparison skills, and mathematical performance relate to each other.

In addition, some researchers believed that the ability to map between symbolic and non-symbolic quantities was an important factor in the development of children’s mathematical ability (Brankaer et al., 2014). This may be because the mapping capability reflects an individual’s ability to process different types of magnitude information. The better one is at mapping, the better he/she could learn advanced mathematics. Mundy and Gilmore (2009) tested children’s bi-directional mapping ability and their mathematical performance. A significant prediction of mapping ability was found for mathematical performance. Similar results were also found by Kolkman et al.’s (2013) and Brankaer et al. (2014) path analyses. However, Friso-van Den Bos et al. (2015) tracked 442 5-year-olds for 3 years; they found children’s mapping skill did not significantly predict their mathematical achievements. Therefore, the impact of mapping skills on mathematical ability has not been uniformly concluded.

### Present Study

In sum, this study aims to achieve two goals. First, we aim to provide detailed development trajectories of non-symbolic and symbolic representation skills in childhood. Previous studies mostly focused a few age groups (Barth et al., 2005, 2006, 2008; Gilmore et al., 2007; Xenidou-Dervou et al.’s 2015). Data capturing a longer developmental period throughout childhood are needed. The available evidence showed both distinctions and connections between symbolic and non-symbolic comparison abilities. We predict that children are more experienced at the non-symbolic task than symbolic task in early childhood, but as they learn more about the symbolic representation system, children’s advantage in non-symbolic skill will disappear. Second, this study aims to investigate the associations between numerical representation skills and mathematical ability in childhood. Researchers investigating the issue focused on different age ranges and therefore generated different results (Gobel et al., 2014; van Marle et al., 2014; Friso-van Den Bos et al., 2015). The exact relations between non-symbolic, symbolic comparison, and mathematical performance remain unclear. We focused the age range of 4 to 8 and predicted that the relationships between these three types of abilities might be different for different age groups in our study.

## Materials and Methods

### Ethics Statement

This research was approved by the local ethical committee of Beijing Normal University. We obtained informed written consent from caretakers or guardians on behalf of the child participants involved in the study, according to the institutional guidelines of Beijing Normal University.

### Participants

A total of 253 children (116 girls) were recruited from 2 public schools located in Baoji, Shaanxi province, China. Forty-six 4-year-olds (*M* = 48.1 months, *SD* = 4.2), 61 5-year-olds (*M* = 59.6 months, *SD* = 3.6), and 62 6-year-olds (*M* = 73.4 months, *SD* = 3.4) were recruited from one kindergarten; 39 7-years-olds (*M* = 83.2 months, *SD* = 2.5) and 45 8-years-olds (*M* = 96.3 months, *SD* = 3.4) were recruited from a primary school (the 1st and 2nd grades). All children were tested around March, during the second half of the Chinese academic year. All children are Mandarin native speakers. They were mostly from families of middle socioeconomic status. All children gave oral consent and their parents gave written consent before participation. A gift (i.e., a book) was sent to each child after participation.

### Measures

#### Number-Naming

Children’s number-naming ability was measured. They were asked to read loudly 50 Arabic numbers, which were written in five lines on a piece of paper (21 cm × 29.7 cm). Numbers on the five lines were 1–10, 11–20, 21–30, 31–40, and 41–50 successively. Children obtained 1 point for successfully naming all numbers in one line. Otherwise, they obtained 0 point. The total scores ranged from 0 to 5.

#### Verbal-Counting

To assess verbal-counting skills, children were asked to count loudly numbers from 1 to 100. These numbers were divided to ten groups (i.e., 1–9, 10–19, 20–29, until 100). They obtained 1 point for successfully counting one entire group. Otherwise, they obtained 0 point. The total scores ranged from 0 to 10.

#### Non-symbolic Comparison

**FIGURE 1:**
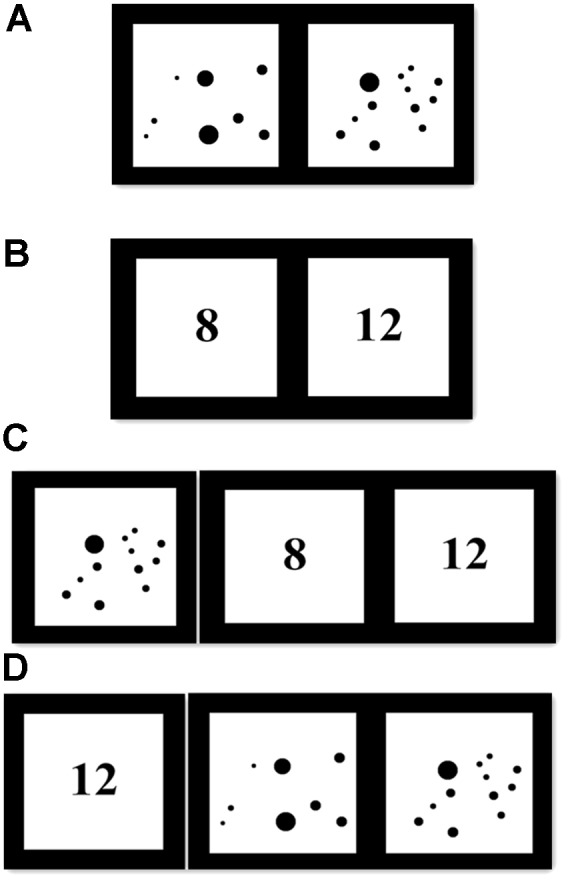
Schematic depictions of numerical comparison tasks and mapping tasks. **(A)** An example trial of non-symbolic comparison task. **(B)** An example trial of symbolic comparison task. **(C)** An example trial of non-symbolic to symbolic mapping task. **(D)** An example trial of symbolic to non-symbolic mapping task.

We tested children’s non-symbolic skills using tasks programmed in E-prime. Similar to Wagner and Johnson (2011), we presented participants two black dots arrays and they were asked to decide, without counting, which one contained more dots (see **Figure 1A**). Children were instructed pressing “C” key for quantity on the left and pressing “M” key for quantity on the right. They had a maximum of 10 s to respond and they were required to respond as accurately and quickly as possible. If children did not respond within the 10 s, the trial would automatically be coded as incorrect. The inter-trial interval was 1000 ms. All children received four practice trials, followed by feedback (“√” or “×”) to make sure they understand the task. After that, they received 32 test trials without feedback.

The numerosities included in this task ranged from 5 to 50. The numerical ratios between the two dot arrays were 2/3, 3/4, 4/5, 5/6. There were eight test trials at each ratio level^1^. The order of test trials was random. The probability of large or small numerosities is balanced on the two sides. The dots were constructed in Microsoft Visual C++ 6.0, with the size ranging from 0.2 to 0.6 cm. To rule out judgments based on the continuous dimension of surface area rather than number, the paired dot arrays were matched for total area filled (Feigenson et al., 2002; Rousselle et al., 2004).

#### Symbolic Comparison

This task was identical to the non-symbolic comparison task except that all dots were replaced by their corresponding Arabic numbers (see **Figure 1B**). Numbers used in each comparison were the same as those in the non-symbolic task. All children received 4 practice trials and 32 test trials.

#### Mapping

We used a similar task to Mundy and Gilmore (2009), which contained two sub-tasks: (1) Non-symbolic to symbolic mapping task (N-S task). In this task, a target dot array was presented, followed by two alternative Arabic numbers (See **Figure 1C**). Children were asked, “Which Arabic number was equal with the previous dot array?” (2) Symbolic to non-symbolic mapping task (S-N task). In this task, a target Arabic number was presented, followed by two alternative dot arrays (See **Figure 1D**). Children were asked, “Which dot array was equal with the previous Arabic number?” similarly, children were asked to press “C” or “M” key to response. The target quantity lasted for 1000 ms and then the alternative choices were presented. Children had a maximum of 10 s to respond and they were required to respond as accurately and quickly as possible. If children did not respond within the 10 s, the trial would automatically be coded as incorrect. The inter-trial interval was 1000 ms. For sub-tasks, children received 4 practice trials and 24 test trials.

The target quantities varied from 5 to 50, and the alternative choices consisted of the correct quantity and a distractor. The ratio between the correct quantity and the distractor were 2/3 and 4/5. There were 12 test trials at each ratio level^2^. The correct quantities were counterbalanced in comparable amount within a pair (i.e., larger or smaller) across trials. The same numerosities were tested in both sub-tasks.

#### Mathematical Competence

We administered Form A of the Test of Early Mathematics Ability-Third Edition (TEMA-3; Ginsburg and Baroody, 2003) to assess their mathematical ability. The TEMA-3 measures many aspects of mathematical performance in childhood, such as numeracy skills (e.g., verbally naming written numbers), number-comparison skills (e.g., determining which of two dot arrays is more), calculation skills (e.g., solving addition or subtraction problems physically or mentally), and number concepts (e.g., answering how many hundreds are in one thousand). It consists of 72 items. Following the standardized administration of the TEMA-3, we started testing with items according the norms for each age group. The test stopped when a child answered 5 consecutive items incorrectly. Scores from the TEMA-3 was normalized for children from 3 years 0 months to 8 years 11 months, and previous research (Ginsburg and Baroody, 2003; Mazzocco et al., 2011) showed relatively high test–retest reliabilities (*r* = 0.82, 0.93) of TEMA-3. Meanwhile, children’s performances on TEMA-3 are also highly correlated with their performances on other math achievement tests (Newcomer, 2001; Woodcock et al., 2001).

### Procedure

Children were tested individually in a quiet laboratory room, accompanied by one experimenter. All participants complete the number-naming and verbal-counting tasks first, and then the non-symbolic, symbolic comparison tasks and mapping task, which were programmed in E-prime version 2.0 (Psychological Software Tools, Pittsburgh, PA, United States) and presented by a Dell E450 computer. Children complete TEMA-3 last. A short break was provided in-between of tasks. Children received a small reward after the experiment.

## Results

### Descriptive Statistics

Four- to 8-year-olds’ performances on the number-naming task, the verbal-counting task, non-symbolic, symbolic comparison tasks, mapping tasks, and TEMA-3 were presented in **Table 1**. One-sample *t*-tests showed that all age groups performed well above chance-level in the non-symbolic comparison task. However, only 5- to 8-year-olds performed above chance in symbolic comparison task. Six- to 8-year-olds performed above chance in mapping tasks, but not 4-to 5-year-olds.

**Table 1:** Children’s performance in numerical comparisons, mapping tasks, and mathematical ability test.

	4 years old		5 years old		6 years old		7 years old		8 years old	
					
	*M*	*SD*	*M*	*SD*	*M*	*SD*	*M*	*SD*	*M*	*SD*
Na	1.600	1.195	3.390	1.715	4.980	0.127	5.000	0.000	5.000	0.000
VC	2.150	1.966	5.440	3.165	9.160	1.883	10.000	0.000	10.000	0.000
N	0.649***	0.158	0.811***	0.169	0.861***	0.126	0.926***	0.096	0.894***	0.133
	*d* = 0.689		*d* = 0.843		*d* = 0.944		*d* = 0.976		*d* = 0.948	
S	0.517	0.053	0.731***	0.161	0.837***	0.138	0.893***	0.098	0.896***	0.084
			*d* = 0.822		*d* = 0.925		*d* = 0.971		*d* = 0.978	
NS	0.511	0.037	0.513	0.135	0.568**	0.147	0.564**	0.141	0.562**	0.142
					*d* = 0.424		*d* = 0.418		*d* = 0.404	
SN	0.502	0.042	0.558	0.171	0.578***	0.159	0.579***	0.122	0.589***	0.150
					*d* = 0.442		*d* = 0.548		*d* = 0.513	
TEMA-3	110.77	7.316	111.43	8.449	108.75	7.534	110.36	9.923	112.53	6.541


*Na, number-naming ability; VC, verbal-counting ability; N, non-symbolic comparison task; S, symbolic comparison task; NS, non-symbolic to symbolic mapping; SN, symbolic to non-symbolic mapping. One-sample *t*-tests were used to compare children’s accuracies in non-symbolic, symbolic comparison tasks, mapping tasks with the chance level, ^∗∗∗^indicates *p* < 0.001, ^∗∗^indicates *p* < 0.01. *d* refers to the effect size.*

### The Development Trajectories of Non-symbolic and Symbolic Representation Abilities

**FIGURE 2:**
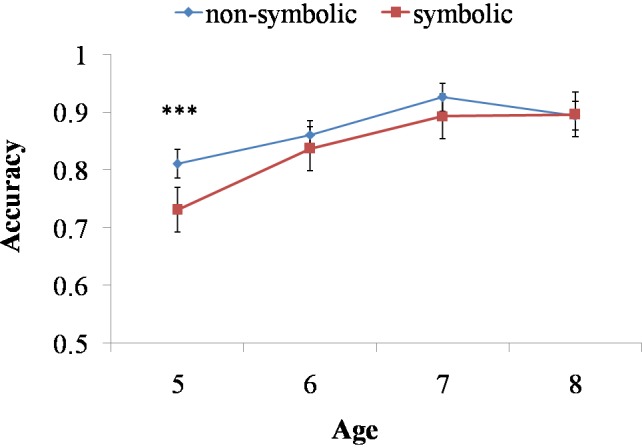
The interaction of age and task across non-symbolic and symbolic comparison tasks. Children performed significantly better in non-symbolic comparison task than the symbolic one at 5 years old. Accuracies on symbolic and non-symbolic comparison tasks were not different for 6-, 7-, and 8-year-olds. ^∗∗∗^Indicates *p* < 0.001.

Four-year-old children performed at chance level in symbolic comparison task. Therefore, their data were eliminated from the following analysis. In order to provide detailed descriptions on the development of non-symbolic and symbolic representation capacities during childhood, we conducted a 2 (Task: non-symbolic and symbolic) × 4 (Ratio: 2:3, 3:4, 4:5, 5:6) × 4 (Age: 5, 6, 7, 8 years old) repeated measures ANOVA on children’s performance accuracy. Mauchly’s test indicated that the assumption of sphericity had been violated for Ratio, χ^2^(5) = 19.256, *p* = 0.002. Therefore, we corrected the degrees of freedom by using the Greenhouse–Geisser estimates. The Box’s *M* test result for the homogeneity of variance hypothesis was significant (Box’s *M* test = 324.071, *F* = 2.742, *p* = 0.000). Therefore, we showed the results of Friedman and Wilcoxon non-parametric test at the same time. Results demonstrated the main effects of Ratio, *F*(2.800,489.916) = 43.220, *p* < 0.001, $\eta_{p}^{2}$ = 0.198, Task, *F*(1.000,175.000) = 11.611, *p* < 0.010, $\eta_{p}^{2}$ = 0.062, Age, *F*(3,175) = 12.312, *p* < 0.001, $\eta_{p}^{2}$ = 0.174, a significant interaction between Task and Ratio, *F*(2.855,504.891) = 19.649, *p* < 0.001, $\eta_{p}^{2}$ = 0.101, and a marginal significant interaction between Task and Age, *F*(3.000,175.000) = 2.639, *p* = 0.051, $\eta_{p}^{2}$ = 0.043. Further simple effect analyses (and the Friedman non-parametric test) for the interaction between Task and Ratio indicated that, both in non-symbolic and symbolic comparison tasks, there was a significant ratio effect, *F*_non-symbolic_(3,525) = 17.720, *p* < 0.001, $\eta_{p}^{2}$ = 0.091 [χ^2^(3) = 68.208, *p* < 0.001], *F*_symbolic_(3,525) = 43.660, *p* < 0.001, $\eta_{p}^{2}$ = 0.199 [χ^2^(3) = 104.614, *p* < 0.001]. Further simple effect analyses for the interaction between Task and Age demonstrated that, 5-year-olds were better at non-symbolic task than symbolic task, *F*(1,175) = 12.910, *p* < 0.001, $\eta_{p}^{2}$ = 0.068, but other age groups performed equally on the symbolic and the non-symbolic task, *F*_6-year-olds_(1,175) = 2.190, *p* = 0.141, *F*_7-year-olds_(1,175) = 2.500, *p* = 0.116, *F*_8-year-olds_(1,175) = 0.010, *p* = 0.914 (See **Figure 2**). The Wilcoxon non-parametric test confirmed the similar effect of age, *Z*_5-year-olds_ = -2.570, *p* < 0.050, *Z*s for other age groups were from -1.504 to -0.296, *P*s > 0.050. These results suggested the advantage of non-symbolic numerical representations over symbolic ones was salient in early childhood. However, after 5, as children learn more about the symbolic representation system, their advantage in non-symbolic representations disappeared.

### The Associations Between Numerical Representation Skills and Mathematical Ability

Correlation coefficients and partial correlation coefficients (controlling for age) between different tasks were presented **Table 2**. There were strong associations between number-naming, verbal-counting skills, non-symbolic and symbolic comparison tasks and mathematical ability, but after controlling for age, the correlations between verbal-counting abilities, numerical comparison skills, and mathematical ability were not anymore significant. This indicated that the verbal-counting ability had no significantly direct effect on non-symbolic, symbolic comparison, and mathematical skills. However, both correlation and partial correlation analyses showed strong associations between number-naming, numerical comparison, and mathematical skills, and between the mapping skills and symbolic representation skills. These close links between each type of skills and the mathematical ability allow us to construct a structure model to better understanding of the mechanism.

**Table 2:** Correlation coefficients and partial correlation coefficients (controlling for age) between different numerical tasks.

		Na	VC	N	S	NS	SN
Na	*r*	1.000					
	*r*_p_	1.000					
VC	*r*	0.867^∗∗∗^					
	*r*_p_	0.440^∗∗∗^					
N	*r*	0.556^∗∗∗^	0.548***				
	*r*_p_	0.218^∗∗^	0.045				
S	*r*	0.555^∗∗∗^	0.251**	0.517^∗∗∗^			
	*r*_p_	0.435^∗∗∗^	0.129	0.465^∗∗^			
NS	*r*	0.055	0.077	0.088	0.155^∗^		
	*r*_p_	0.060	0.069	0.091	0.163^∗^		
SN	*r*	0.086	-0.026	0.109	0.198^∗∗^	0.069	
	*r*_p_	0.084	-0.039	0.104	0.204^∗∗^	0.069	
TEMA-3	*r*	0.809^∗∗∗^	0.727***	0.570^∗∗∗^	0.568^∗∗∗^	0.118	0.087
	*r*_p_	0.296^∗∗∗^	0.044	0.228^∗∗^	0.426^∗∗∗^	0.185^∗^	0.108


*^∗^Indicates *p* < 0.050, ^∗∗^indicates *p* < 0.010, ^∗∗∗^indicates *p* < 0.001. Na, number-naming ability, VC, verbal-counting ability; N, non-symbolic comparison task; S, symbolic comparison task; NS, non-symbolic to symbolic mapping; SN, symbolic to non-symbolic mapping.*

We conducted structural equation modeling (SEM) analyses to examine the associations between non-symbolic, symbolic, mapping skills, and mathematical ability using Mplus Version 7. We developed one model for the developmental period from age 5–8 (Model A) and four separate models for each age groups (see **Table 3**, Model B was for 5-year-olds, Model C was for 6-year-olds, Model D was for 7-year-olds, Model E was for 8-year-olds). The SEM fit indexes (Confirmatory Fit Index and Root Mean Square Error of Approximation) suggested a goodness of fit for all five models (see **Table 3**). Model A, capturing the entire developmental period from age 5 to 8, explained 42.1% of the variance in mathematical ability. It revealed a direct effect of symbolic skills on mapping skills and mathematical ability (see the effect values marked in **Table 3**). Children’s non-symbolic skills affected their mathematical ability indirectly, via symbolic skills. Comparing the four models for different age groups, we found that this indirect effect of non-symbolic skills on mathematical ability was only significant for 5- and 6-year-olds, but not for 7- and 8-year-olds. The direct effect of symbolic skills on mathematical ability was significant for 5-, 6-, and 7-year-old, but not for 8-year-olds. Furthermore, the effect values of both non-symbolic and symbolic numerical representation skills on mathematical performance declined with age (see effect values marked in **Table 3**). Across models, we did not found significant effects of mapping skills on mathematical ability.

**Table 3:** The SEM of non-symbolic, symbolic representation, mapping skill, and mathematical ability.

Model A (5- to 8-year-olds) Bootstrap: χ^2^(1) = 0.379, *p* = 0.538 CFI = 1.000 RMSEA = 0.000	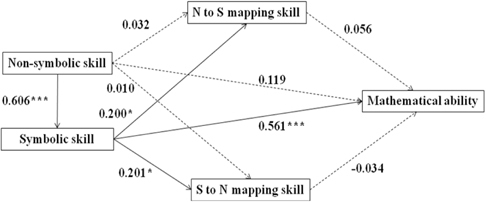
	SEM analyses revealed the indirect effect value of the non-symbolic number skills on the mathematical ability is 0.340 (*p* < 0.000).
Model B (5-year-olds) Bootstrap: χ^2^(1) = 0.130, *p* = 0.719 CFI = 1.000 RMSEA = 0.000	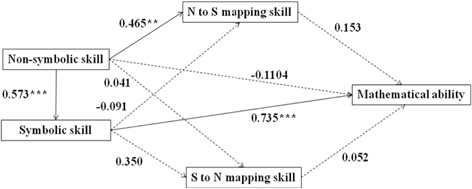
	SEM analyses revealed the indirect effect value of the non-symbolic number skills on the mathematical ability is 0.421 (*p* < 0.010).
Model C (6-year-olds) Bootstrap: χ^2^(1) = 0.083, *p* = 0.773 CFI = 1.000 RMSEA = 0.000	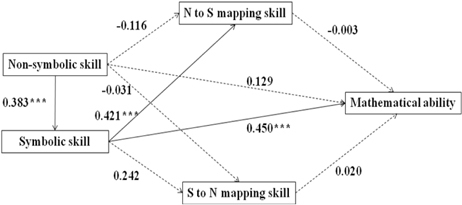
	SEM analyses revealed the indirect effect value of the non-symbolic number skills on the mathematical ability is 0.172 (p < 0.050).
Model D (7-year-olds) Bootstrap: χ^2^(1) = 2.563, *p* = 0.109 CFI = 0.950 RMSEA = 0.053	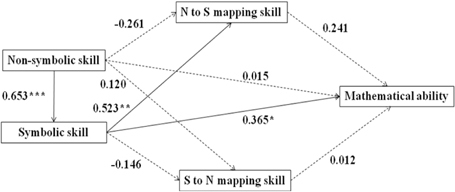
	SEM analyses revealed the indirect effect value of the non-symbolic number skills on the mathematical ability is not significant. The indirect effect value is 0.238 (*p* = 0.063).
Model E (8-year-olds) Bootstrap: χ^2^(1) = 0.014, *p* = 0.906 CFI = 1.000 RMSEA = 0.000	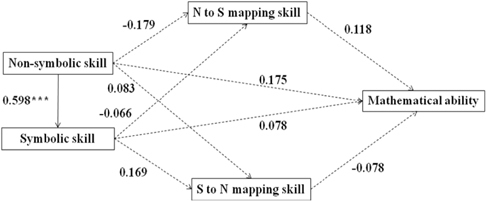
	SEM analyses revealed the indirect effect value of the non-symbolic number skills on the mathematical ability is not significant. The indirect effect value is 0.046 (*p* = 0.672).


*N to S mapping skill, Non-symbolic to Symbolic mapping skill; S to N mapping skill, Symbolic to Non-symbolic mapping skill. ^∗^Indicates *p* < 0.05, ^∗∗^indicates *p* < 0.01, ^∗∗∗^indicates *p* < 0.001.*

## Discussion

We investigated two issues in our study. First, we showed detailed developmental trajectories of non-symbolic and symbolic representation skills from age 4 to 8. Children were able to do non-symbolic representation task at age 4. Five-year-olds performed better in the non-symbolic task than they did in the symbolic one. However, after 5, as children learn more about the symbolic representation system, their advantage of non-symbolic skills disappeared. Second, we found a significant effect of symbolic skills on math performance and an indirect effect of non-symbolic skills on the mathematical ability via symbolic skills. Both the direct effect of symbolic skills and the indirect effect non-symbolic skills declined with age. This suggests that non-symbolic and symbolic numerical representation skills may no longer be the major factors for math performance of children in primary school.

### The Developmental Trajectories of Non-symbolic and Symbolic Representation Abilities

A variety of studies suggested the inherent and universal nature of non-symbolic representation (Wynn, 1992; Pica et al., 2004; Flombaum et al., 2005). The current study demonstrated children as young as 4 years old were able to represent and compare non-symbolic quantities of range 5 to 50 successfully and flexibly. Similar paradigm was also used by Toll et al.’s (2015) testing children’s non-symbolic comparison for numbers ranging from 1 to 100. Children performed well on their non-symbolic comparison task starting from age 4. For a smaller and narrower range of number from 4 to 15, researchers found similar results in 4-year-olds (Libertus et al., 2011). Wagner and Johnson (2011) assessed non-symbolic comparison skills with numbers range 1–50. They found 3-year-olds performed above chance level in non-symbolic comparison task with numerosities 1–4. To prevent children from precisely tracking dots, we used numerosities larger than 4. Although different stimuli were used in our study, the present results are still in line with previous studies, which provided evidence for the development of non-symbolic capacity after age 4 (Libertus et al., 2013; Vanbinst et al., 2015). However, for symbolic representation, our study showed 5-year-olds and older children, but not 4-year-olds, performed well in our comparison task. Similarly, previous studies (Gilmore et al., 2007; Kolkman et al., 2013) reported that children started being able to do symbolic representation task at the age of 5, before the start of formal schooling. Furthermore, researchers had found symbolic representation skills developed continuously during childhood (Li et al., 2017). These results indicate the acquired nature of the symbolic comparison skills. As a learned ability, its development is built on some more fundamental capacities, such as non-symbolic representations. Our SEM analyses showed a significant effect of non-symbolic skills on symbolic skills (see effect values in model B to model E). The indirect effect of the non-symbolic skills on mathematical abilities was carried out by symbolic skills. Therefore, we think, to some extent, the mastery of non-symbolic comparison skills was as precondition for the development of symbolic comparison skills.

There are limited studies in the field describing development trajectories of non-symbolic and symbolic comparison ability for a larger age span in childhood. Oftentimes researchers only investigated 2 to 3 age groups. For example, Xenidou-Dervou et al. (2015) focused on 5- and 6-year-olds. They also considered the developmental changes of non-symbolic and symbolic abilities. However, they used the approximate addition tasks, which were more difficult than the approximate comparison tasks in our study. In their task, children had to add the two quantities first and then to compare the numerosities, which required the arithmetic ability at the same time. Xenidou-Dervou et al. (2015) found that the ability of symbolic addition emerged around age 6. Our results provide detailed developmental trajectories of non-symbolic and symbolic comparison abilities for a larger age span in childhood. We found that 4-year-olds were able to do non-symbolic comparisons, but not symbolic comparisons. Five-year-olds were able to do both types of comparisons, but they performed better at the non-symbolic task than the symbolic one. However, this performance difference disappeared around the age of six. We think these developmental changes may be related to the different characteristics of non-symbolic and symbolic skills. Non-symbolic representation ability is inherent, shared by humans and animals (Wynn, 1992; Pica et al., 2004; Flombaum et al., 2005). However, symbolic comparison ability is affected by education (Xenidou-Dervou et al., 2015), and its emergence requires a certain foundation (Kolkman et al., 2013). Many researchers have found that children’s symbolic representation skill will rapidly increase in the 1st grade (Xenidou-Dervou et al., 2015; Li et al., 2017). Therefore, we observed that children could pass non-symbolic tasks at a very young age, but they were not able to pass symbolic representation tasks until 5 years old. However, with more education, children’s symbolic skills improve rapidly and their advantage in non-symbolic skills disappears around 6 years old.

### The Associations Between Numerical Representation Skills and Mathematical Ability

Fazio et al. (2014) proposed three hypotheses about the relationship between non-symbolic, symbolic skills, and mathematical ability: (1) non-symbolic skills have indirect effects on mathematics achievement. That is, children with better non-symbolic skills acquire the symbolic numerical system more easily, which in turn improves their mathematical ability; (2) non-symbolic skills have both direct and indirect effects on mathematics achievement; (3) non-symbolic and symbolic skills may independently affect overall mathematics achievement. In the current study, we found an indirect effect of non-symbolic skills on mathematical abilities via symbolic skills, which supports Fazio et al.’s (2014) first hypothesis. Similar results were also found by van Marle et al. (2014), who assessed non-symbolic, symbolic skills, and mathematics achievement in 4-year-olds and found that the relation between non-symbolic skills and mathematics achievement was fully mediated by children’s symbolic skills. Differently, a significant positive correlation between the precision of non-symbol quantity and mathematical achievement in 3- to 5-year-old children was reported by Libertus et al. (2012). They used children’s ANS acuity, rather than accuracy, as an indicator of children’s non-symbolic skill. The ANS acuity is represented by Weber’s fraction, which is derived from the theoretical hypothesis of psychophysics. It is an indirect indicator for numerical representation ability. However, the ANS accuracy illustrates numerical representation ability more directly. This measurement difference might result the different findings here. On the other hand, as shown in previous studies (Kolkman et al., 2013; Toll et al., 2015), we also found a significant effect of symbolic skills on mathematical ability.

In addition, we found that children’s mapping ability had no significant effects on their mathematical ability. However, using similar paradigm, Mundy and Gilmore’s (2009) found children’s bi-directional mapping ability predicted their mathematical achievement significantly. This result might be because, comparing to our tasks using comparison ratios of 2/3 and 4/5, Mundy and Gilmore’s (2009) tasks were easier. They used relative easy comparison ratios of 1/2 and 2/3. Other researchers used different paradigms to assess children’s mapping ability. For example, Kolkman et al. (2013) found mapping skills was an important predictor for math performance. However, they used symbolic number-lines and symbolic comparison tasks, which are very different from our bi-directional mapping task. Therefore different results were generated.

Finally, we found the associations between numerical representation skills and mathematical abilities varied across age groups. The indirect effect of non-symbolic skills on mathematical abilities was only significant for 5- and 6-year-olds, but not for 7- and 8-year-olds. The direct effect of symbolic skills on mathematical abilities was significant for 5-, 6-, and 7-year-olds, but not for 8-year-olds. In general, the impacts of non-symbolic and symbolic numerical representation skills on mathematical performance both declined with age. We think the result may suggest, with age, non-symbolic, and symbolic numerical representation skills were no longer major factors for math performance. Similar developmental trend has been found in previous studies as well. A significant positive correlation between the non-symbolic skill and mathematical achievement was reported for 3- to 5-year-olds (Libertus et al., 2012); with age, this positive correlation disappeared for 6- to 8-year-olds (Holloway and Ansari, 2009). Meanwhile, there are studies (Halberda et al., 2008; Bugden and Ansari, 2011; Linsen et al., 2015) showed correlations between numerical representation skills and mathematical ability throughout childhood. However, their methods were quite different from ours. For example, instead of TEMA-3, Bugden and Ansari (2011) used two mathematics subtests from the Woodcock Johnson III and Linsen et al. (2015) used multi-digit subtraction task to assess children’s mathematical ability. Also, many of previous studies only investigated 2 to 3 age groups, which may affect how their results can be generalized.

### Limitations and Future Research

The current study has limitations and therefore requests future research to further clarify these questions. First, with the cross-sectional design of the current study, the developmental information provided by the data was limited. We were not able to examine longitudinally interactions of non-symbolic and symbolic representation skills and their association with mathematical ability. This requests future research to clarify the issue. In fact, we are currently working on the follow-up of this study. With the longitudinal data, we would be able to draw a more comprehensive picture on the development of children’s numerical representation capacities and their association with mathematical performance. Second, in this study, we only considered numerosities larger than 4, which made tasks difficult for 4-year-olds. The reason we used numerosities larger than 4 is to prevent children from precisely tracking dots, because previous research (Feigenson et al., 2004) shown that children developed a system to keep track of small numbers precisely from very young. However, with numerosities smaller than 4, we may be able to capture 4-year-olds’ performance in the symbolic comparison task. Future research needs to address this issue and compare children’s non-symbolic and symbolic comparison skills and mapping ability for both large and small numerosities.

## Author Contributions

YL contributed to conception and design, on acquisition and interpretation of data, and on drafting of manuscript. YC contributed to conception and design and on interpretation of data. MZ contributed to drafting of manuscript. XZ and ZD contributed on interpretation of data. SY contributed to making the experimental materials and entering the data.

## Conflict of Interest Statement

The authors declare that the research was conducted in the absence of any commercial or financial relationships that could be construed as a potential conflict of interest.

## Footnotes

1 The paired arrays tested for ratio 2/3 were 6 vs. 9, 8 vs. 12, 10 vs. 15, 12 vs. 18, 14 vs. 21, 16 vs. 24, 18 vs. 27, and 20 vs. 30. The paired arrays tested for ratio 3/4 were 6 vs. 8, 9 vs. 12, 12 vs. 16, 15 vs. 20, 18 vs. 24, 21 vs. 28, 24 vs. 32, and 27 vs. 36. The paired arrays tested for ratio 4/5 were 8 vs. 10, 12 vs. 15, 16 vs. 20, 20 vs. 25, 24 vs. 30, 28 vs. 35, 32 vs. 40, and 36 vs. 45. The paired arrays tested for ratio 5/6 were 5 vs. 6, 10 vs. 12, 15 vs. 18, 20 vs. 24, 25 vs. 30, 30 vs. 36, 35 vs. 42, and 40 vs. 48.

2 The pairs tested for ratio 2/3 were 6 vs. 9, 8 vs. 12, 10 vs. 15, 12 vs. 18, 14 vs. 21, and 16 vs. 24. The correct quantities were 9, 12, 10, 18, 14, and 16, respectively. The pairs tested for ratio 4/5 were 8 vs. 10, 12 vs. 15, 16 vs. 20, 20 vs. 25, 24 vs. 30, and 28 vs. 35. The correct quantities were 8, 15, 16, 25, 24 and 35, respectively. Each pair was tested twice.
